# Whole genome scanning and association mapping identified a significant association between growth and a SNP in the IFABP-a gene of the Asian seabass

**DOI:** 10.1186/1471-2164-14-295

**Published:** 2013-05-01

**Authors:** Jun Hong Xia, Grace Lin, Xiaoping He, Peng Liu, Feng Liu, Fei Sun, Rongjian Tu, Gen Hua Yue

**Affiliations:** 1 Molecular Population Genetics Group, Temasek Life Sciences Laboratory, 1 Research Link, National University of Singapore, 117604 Singapore, Singapore

**Keywords:** Single nucleotide polymorphism, Growth trait, Candidate gene, Quantitative trait locus

## Abstract

**Background:**

Aquaculture is the quickest growing sector in agriculture. However, QTL for important traits have been only identified in a few aquaculture species. We conducted QTL mapping for growth traits in an Asian seabass F_2_ family with 359 individuals using 123 microsatellites and 22 SNPs, and performed association mapping in four populations with 881 individuals.

**Results:**

Twelve and nine significant QTL, as well as 14 and 10 suggestive QTL were detected for growth traits at six and nine months post hatch, respectively. These QTL explained 0.9-12.0% of the phenotypic variance. For body weight, two QTL intervals at two stages were overlapped while the others were mapped onto different positions. The IFABP-a gene located in a significant QTL interval for growth on LG5 was cloned and characterized. A SNP in exon 3 of the gene was significantly associated with growth traits in different populations.

**Conclusions:**

The results of QTL mapping for growth traits suggest that growth at different stages was controlled by some common QTL and some different QTL. Positional candidate genes and association mapping suggest that the IFABP-a is a strong candidate gene for growth. Our data supply a basis for fine mapping QTL, marker-assisted selection and further detailed analysis of the functions of the IFABP-a gene in fish growth.

## Background

Growth traits are the major interest in aquaculture. Growth traits are complex traits that regulated by many loci that have quantitative effects on the phenotype. Due to the ease of measurement, the economic importance and intermediate heritabilities for growth traits, phenotypic selection has been commonly and effectively applied in fish breeding programs. However, the process of developing new varieties or lines is labor intensive, time consuming and costly by using traditional breeding methods. Marker-assisted selection (MAS) by using markers tightly linked to quantitative trait loci (QTL) as a substitute to assist phenotypic screening, can overcome the shortcomings in traditional breeding, thus increasing the accuracy and efficiency of selection [1]. This technique is especially valuable for traits with low to moderate heritability, which are difficult to be improved by traditional selection.

Identification of QTL related to growth is the basis for application of genetic markers in fish breeding. A key step in QTL analysis of complex traits is the establishment of large collections of genetic markers [2]. A few studies are available on linkage and QTL mapping for growth traits in fish, such as salmonids [3-6], sea bream [7-11], rainbow trout [12], tilapia [13,14], Asian and European seabass [15-20] and turbot [21]. However, the identification of QTL underlying complex traits have proved to be very challenging due to gene-gene and gene-environment interactions, low proportion of explained phenotypic variation for each locus [22,23], the large number of QTL, and many additional sources of variation [24]. Linkage-based QTL mapping and linkage disequilibrium (LD)-based genetic association mapping are two major approaches to locate genes that control phenotypes of interest. Combined linkage and association analysis showed significant improvement in detection of QTL and have shown the ability to unravel the secrets of complex traits in animal breeding [25]. Few such reports exist for fish to date.

Many traits with importance in agriculture change with time or other independent variables [26]. In animal, many factors such as skeletal structure, organ, muscle, and fat mass contribute to overall body size [27] and family-based, age-related and strain-specific growth differences are likely to co-exist [28-30]. Genetic factors affecting growth are expected to vary among individuals, families and different stages of development. The power of QTL analysis is therefore seriously limited, since only segregating QTL in one or both parents that were used in constructing mapping families can be detected [21]. Although some family/population-specific QTL have been reported in the Asian seabass [15-17] and other fishes [28-30], the molecular and genetic basis of growth traits remains fragmentary, highlighting the need for additional studies of these important trait in fish.

The Asian seabass *Lates calcarifer* is an important farmed marine foodfish species, widely distributed in the Indo-West Pacific region (FishBase: http://www.fishbase.org). Currently a second generation linkage map for the Asian seabass was developed with 790 markers [15]. Based on the genetic map information, some QTL for growth traits were mapped [15-17]. Significant QTL for growth traits measured at the age of 3 months were mapped onto linkage groups 2 and 3 [16,17] using F_1_ families. Fine mapping of QTL on linkage groups 2 and 3 identified a QTL cluster underlying growth traits in the Asian seabass [15]. These studies provided preliminary data for application of MAS selection in the Asian seabass. However, due to the low analytic power of F_1_ families for QTL mapping, some QTL for growth might be missed.

The potentials of MAS to improve traits are increasingly evident in aquaculture. Identification of QTL-related economic traits is a major aim of fish genome research. In Southeast Asia, the harvesting time for the aquacultured Asian seabass is starting from about nine months of age. In order to map QTL for growth traits at different farming stages of the species, we conducted a whole genome scan for growth traits at the age of six and nine months post hatch by using an F_2_ family including 359 individuals, and genotyping with 123 microsatellites and 22 SNPs. We identified significant and suggestive QTL that affect growth-related traits (body weight, standard length and total length measured at the age of 6 and 9 months) in the Asian seabass. In addition, we detected SNPs in 19 candidate genes related to growth, nutritional condition or environmental adaptation and mapped them in the linkage map of Asian seabass. The gene IFABP-a located on LG5 was significantly associated with growth. Our study provides a basis for identification of genes controlling growth traits and conducting of MAS in the Asian seabass.

## Results and discussion

### Phenotypic data in the F_2_ mapping family

In the Asian seabass, QTL mapping for growth traits that measured at the age of 3 months have been reported [15-17]. Significant QTL for growth traits were mapped onto linkage groups 2 and 3 [16,17]. In these previous studies, only F_1_ families were used in QTL mapping. Due to the low power of F_1_ families for QTL mapping, some QTL might not be detected. To identify QTL that have not been identified in previous studies, we carried out a QTL analysis for growth traits that measured at the age of 6 and 9 months in an F_2_ full sib mapping family (F2S2; N = 359) of the Asian seabass. Body weight, standard length and total length data (named as BW6M, BW9M, SL6M, SL9M, TL6M and TL9M, respectively) were measured at two different stages of development. The means (with standard deviation, sd) for the 6 growth traits in the F_2_ population are provided in Table 1. Significant phenotypic differences were observed among individuals of the family. The observed phenotypic values ranged from 44 g to 442 g (~10 fold differences) with an average of 227.6 (± 62.0 sd) g for the trait BW6M, and the values ranged from 117 g to 672 g (~6 fold differences) for the trait BW9M. The phenotypic variance within a population is the result of genetic sources and/or environmental sources. Under the same conditions, a larger phenotypic variance in a population suggests a higher degree of genetic variation. The population F2S2 showed substantial levels of phenotypic variation, therefore, had enough statistical power for QTL detection. Strong positive phenotypic correlations between pairwise traits were observed. The coefficients ranged from 0.73 between BW9M and SL6M to 0.96 between TL9M and SL9M, underlying a common genetic basis for these traits. Similar results were reported in tilapia [13] and turbot [21].

**Table 1:** Identified QTL affecting growth traits in the Asian seabass

**Traits**	**Average (sd)**	**QTL ID**	**Significance level**	**Linkage group**	**QTL interval**	**Peak position (cM)**	**PVE (%)**
BW6M	227.6 (±62.0) g	bw6m_a	*	LG2	15.0-20.0	18.0	3.0
		bw6m_b	***	LG5	0.0	0.0	7.2
		bw6m_c	*	LG7	6.9-21.9	10.9	3.2
		bw6m_d	*	LG11	7.0-8.7	7.7	2.9
		bw6m_e	*	LG13	0.0-2.0	0.0	2.5
		bw6m_f	*	LG21	0.0-5.0	0.0	3.7
		bw6m_g	**	LG23	20.9-34.0	20.9	4.3
		bw6m_h	***	LG24	0.0-5.5	2.0	5.2
BW9M	340.8 (±110.3) g	bw9m_a	***	LG5	0.0	0.0	10.2
		bw9m_b	*	LG7	6.9-23.6	10.9	1.7
		bw9m_c	*	LG9	0.0	0.0	6.2
		bw9m_d	*	LG9	14.8-19.9	17.9	6.0
		bw9m_e	*	LG18	9.2-30.3	17.2-18.2	6.8
SL6M	206.1 (±20.3) cm	sl6m_a	*	LG3	6.9-19.9	12.9-13.9	4.3
		sl6m_b	***	LG5	0.0	0.0	7.2
		sl6m_c	*	LG7	0.0-23.6	15.9	3.8
		sl6m_d	**	LG11	6.0-8.7	7.7	4.6
		sl6m_e	*	LG15	24.7-65.0	33.0	3.0
		sl6m_f	*	LG18	10.2-14.2	11.2-13.2	3.3
		sl6m_g	**	LG21	0.0-5.0	0.0	4.8
		sl6m_h	**	LG24	0.0-5.5	3.0	4.0
		sl6m_i	*	LG24	7.1-8.1	7.1	10.3
SL9M	229.5 (±24.1) cm	sl9m_a	***	LG5	0.0	0.0	2.1
		sl9m_b	*	LG5	36.1-42.5	42.5	10.5
		sl9m_c	*	LG6	4.7-34.2	31.1-33.1	6.4
		sl9m_d	*	LG7	9.9-15.9	12.9-13.9	2.2
		sl9m_e	***	LG9	8.8-21.9	16.9	5.0
		sl9m_f	***	LG15	0.0-85.8	50.0-51.0	11.1
		sl9m_g	*	LG18	2.2-16.2	9.2	3.2
		sl9m_h	***	LG21	0.0-5.0	1.0	7.6
TL6M	238.9 (±22.2) cm	tl6m_a	***	LG5	0.0	0.0	9.3
		tl6m_b	*	LG7	6.9-23.6	12.9	3.8
		tl6m_c	***	LG11	6.0-8.7	7.7	4.8
		tl6m_d	*	LG13	0.0-5.2	0.0-1.0	2.9
		tl6m_e	**	LG15	23.7-73.0	33.0-34.0	4.4
		tl6m_f	*	LG18	0.0	0.0	2.1
		tl6m_g	***	LG21	0.0-5.0	0.0	5.3
		tl6m_h	***	LG24	0.0-5.5	3.0-4.0	4.8
		tl6m_i	*	LG24	7.1-10.1	8.1	12.0
TL9M	277.8 (±28.0) cm	tl9m_a	*	LG2	16.0-21.0	18.0-19.0	5.7
		tl9m_b	***	LG5	0.0	0.0	5.4
		tl9m_c	*	LG7	6.0-17.9	8.9-9.9	0.9
		tl9m_d	**	LG9	9.8-21.9	16.91	1.1
		tl9m_e	***	LG15	23.7-85.8	50.0-54.0	11.0
		tl9m_f	***	LG21	0.0-5.0	1.0	11.2

*, Chromosome-wide LOD significance (*P* < 0.05); **, genome-wide significance (*P* < 0.05); ***, genome-wide significance (*P* < 0.01); PVE: the proportion of phenotypic variance explained. ‘BW6M and BW9M’, body weight at six and nine months; ‘TL6M and TL9M’, total length at six and nine months; ‘SL6M and SL9M’, standard length at six and nine months.

### Linkage and QTL mapping

One hundred and twenty-three microsatellite markers showed informativeness in the F_2_ mapping family. These markers distributes evenly on the 24 linkage groups that defined by Wang et al. [15]. In addition, 22 SNP markers that developed from genes related to growth, nutritional condition, or environmental adaptation were also applied in map construction. Primer information for the SNP markers was listed in Additional file 1: Table S1. Using linkage analysis and LOD ≥ 3 as threshold for mapping data, all loci were finally assigned to 24 linkage groups (LGs), designated as LG1 – LG24, corresponding to the linkage group names of the high-resolution linkage maps [15,16]. The largest linkage group LG17 obtained in this study containing 8 markers and had a length of 93.5 cM, and the shortest linkage group LG20 consisted of 5 markers with a length of 2.3 cM. The total length for the map was 812 cM with an average linkage group length of 33.8 (± 22.1 sd) cM. The average spacing between adjacent markers was 7.8 (± 5.5 sd) cM, therefore meeting the minimum requirements for QTL analysis [31]. Most markers mapped in the same linear order as in the high-resolution linkage maps [15,16]. The differences between two maps could be caused from the differences in mapping population sizes and marker numbers, genotyping errors and the nature of the population, e.g., differences in family structures and female/male specific recombination rates. Summary of linkage map information and the averaged linkage map are presented in Additional file 2: Table S2 and Additional file 3: Figure S1, respectively.

**Figure 1:**
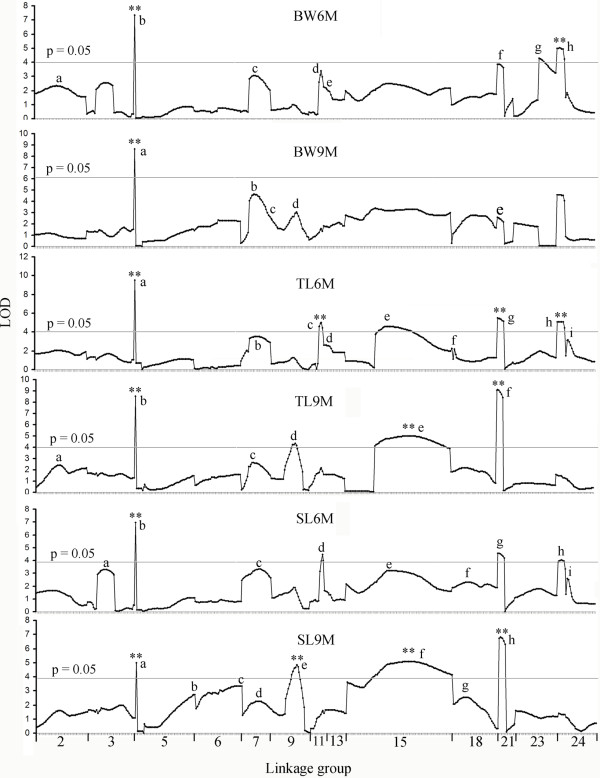
**QTL effects and LOD scores of the Asian seabass genome estimated by QTL mapping.** QTL that surpassed the suggestive LOD threshold (LG-wide threshold: *p* < 0.05) were presented. The lower case alphabet letter sets indicate the identified QTL position in each linkage group for each trait, described as in Table 1. The ‘x’ axis shows the linkage groups with suggestive QTL. The length of each linkage group is roughly proportional to the genetic length (cM) of the respective linkage group. LOD threshold for each trait at genome-wide LOD significance (*p* = 0.05) is shown; ‘**’, highly significant QTL at genome-wide LOD significance (*p* < 0.01).

We analyzed six types of growth traits. Genome-wide (GW) and linkage group (LG)-wide LOD threshold (*p* < 0.01 and 0.05) were calculated from 10000 permutations of the quantitative trait data. Forty-five suggestive QTL including 21 significant QTL (GW threshold: *p* < 0.05) were detected across all traits, which might be representative of the genetic architecture of growth traits in the Asian seabass. Two QTL intervals were detected on each of the 4 linkage groups (LG5, 9, 18 and 24, respectively). No significant association was found in 11 linkage groups (LG1, 4, 8, 10, 12, 14, 16, 17, 19, 20 and 22), probably due to the low informativeness of involved markers and the nature of the genetic architectures of these traits. For each trait, 5 (BW9M) to 9 (TL6M and SL6M) suggestive QTL were detected, suggesting that these traits are highly polygenic. One QTL confidence interval on linkage group LG15 for SL6M and SL9M was large, covering nearly the entire chromosomes. Of the 21 significant QTL (GW threshold: *p* < 0.05), 15 were detected at the genome-wide significant level (GW threshold: *p* < 0.01). QTL that surpassed the suggestive LOD threshold are summarized in Table 1 and shown in Figure 1.

The proportions of phenotypic variance explained (PVE) by all the suggestive QTL were generally small. The highest PVE was 12.0% for the suggestive QTL tl6m_i, which were located on LG24 (8.07 cM). The PVE for the eight QTL of the trait BW6M ranged from 2.5% (bw6m_e) to 7.2% (bw6m_b); and for the nine QTL of trait SL6M, the PVE ranged from 3% (sl6m_e) to 10.3% (sl6m_i). The small PVEs of the QTL that identified per trait suggest that the expression of these traits is under the control of a number of genes. Similar results were obtained in turbot by Sánchez-Molano [21], in which, the PVE of the tightest linked markers to the significant QTL varied from 8.0% to 13.6% for weight and from 8.2% to 12.9% for body length. Some potential candidate genes were detected near or on some of the QTL intervals, e.g. IG2F2BP3 on LG3, IFABP-a on LG5, 2-TNNL2 on LG6, GCDH on LG11, SP on LG13 and APOCI on LG15.

Significant differences in QTL positions for different growth traits were also found, such as sl6m_a on LG3, sl9m_b on LG5 and sl9m_c on LG6, suggesting that different sets of genes are involved in the control of these traits. In a previous study of the Asian seabass [15,16], five significant QTL controlling growths were detected on LG2 and LG3, and explained 6.4 - 59.7% of the observed phenotypic variance, respectively. We also identified two QTL regions on these linkage groups. But, low PVEs and only suggestive significance level (*p* < 0.05) were found for these QTL. These differences could result from the growth variations at different development stages among different families, since we measured traits of fishes at the age of 6 and 9 months, but those used by Wang et al. [15-17] were measured at the 3 months. Specific QTL for body weight, standard length and total length that detected at different sampling time period suggest that there were dynamic expression for the traits, as in salmonids [32], in which some QTL were differentially expressed at different developing stages.

Co-localization of the detected QTL among traits is evident, indicating that pleiotropic effects may be playing a role in growth of the Asian seabass. This result was expected since high genetic correlations among growth traits were found in the seabass and other fish species. All suggestive QTL were located in 17 different genomic regions of 13 linkage groups. QTL regions having similar LOD profiles for multiple traits were detected on 10 linkage groups (LG2, 5, 7, 9, 11, 13, 15, 18, 21 and 24). Interestingly, one QTL region on linkage group LG5 (0~1 cM) was significantly associated to all traits (GW threshold: *p* < 0.01). Given that the high and sharp LOD peak, one common locus might be responsible for the different traits. The PVE of traits for this QTL ranged from 5.4% (tl9m_b) to 10.5% (sl9m_b). Near or on this QTL, eight markers including two SNP markers were located within 1.2 cM regions (Additional file 3: Figure S1 and Figure 1). Interestingly we found IFABP-a-SNP1245 marker showed a significant association to the traits. This result suggests that IFABP-a gene is of great importance in growth of fish and hence, warrant further studies.

### Association of heterozygosity with growth traits in the full sib mapping family

**Figure 2:**
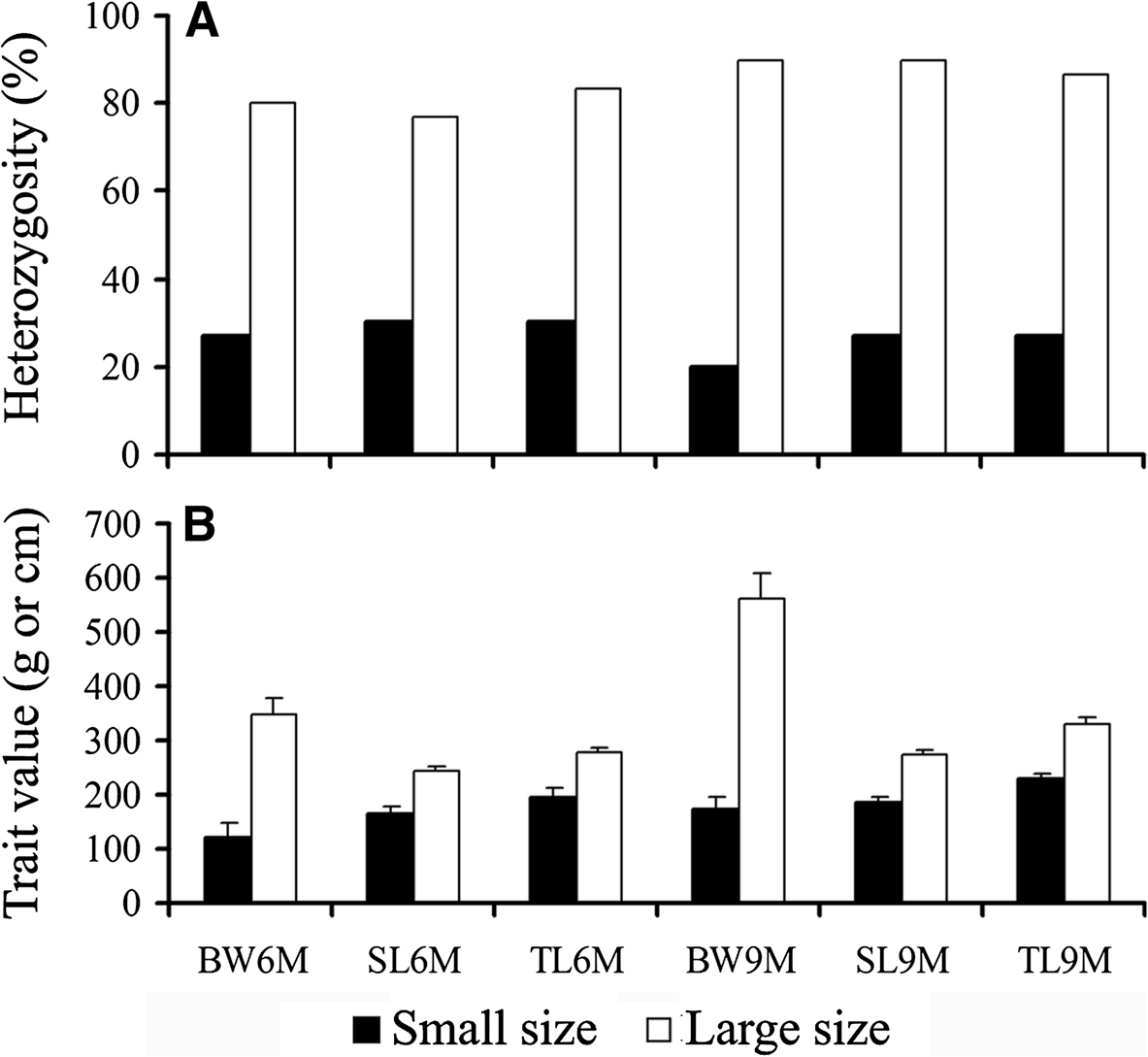
**Heterozygosity effect of the IFABP-a-SNP1245 marker on the growth traits in F2S2 QTL family.** The distribution of heterozygous genotypes (**A**) and observed phenotypic data (**B**) in pairs of extreme size classes (N = 30 for each class) for each trait was provided.

The degree of heterozygosity was used to test for direct effects of the markers on the growth traits. To compare the distribution of genotypes between pairs of extreme size classes for each trait (N = 30 for each class), the degree of heterozygosity for used loci in two extreme size groups of each trait were calculated and tested by using Fisher's exact test. Pairwise tests revealed significant heterogeneity of genotypes among two extreme size groups in multiple markers (Additional file 4: Table S3). The degrees of heterozygosity for 4 loci (SL9M) to 12 loci (SL6M, TL6M) were significantly different between two extreme size groups of each trait. Three SNP markers (IFABP-a-SNP1245, ECHS1, APOE-SNP1) showed significant heterogeneity of genotypes among size-group pairs. The heterozygosities for four of the six loci for BW6M and five of the eight loci for BW9M were highly present in the large size group (80-97% and 37-93%, respectively). The remaining loci show high heterozygosities in the small size group. IFABP-a-SNP1245 show high heterozygosity (CG genotype) in the large size group (77-90%) and show low heterozygosity in the small size group (20-30%) for all six traits (*p* < 0.001) (Figure 2). This result confirmed the putative association of IFABP-a-SNP1245 with growth traits that obtained in QTL analysis. The heterozygosities of marker genotypes that significantly associated with positive effects in traits suggest possible heterosis and potential utility for MAS in the Asian seabass. For example, selection with heterozygote would favor growth in the population. The significant associations between the degree of heterozygosity of loci and traits are presented in Additional file 4: Table S3.

### Analysis of associations between candidate markers and growth traits in 4 populations

The large impact of markers/genes detected in the QTL model does not necessarily mean that the polymorphisms are causative mutations. LD-based association mapping represents a much greater precision than QTL mapping [33]. To verify the association of the markers to body weight that were identified by QTL mapping and display their applications in other families in breeding programs, six markers on or near QTL regions were selected and applied in association analysis of 4 populations produced by mass crosses. These markers were distributed on 5 linkage groups. Two SNP markers (IFABP-a-SNP1245 and IFABP-a-SNP1550) were located on the same gene of linkage group LG5, the other 4 markers located on separate linkage groups. Except the microsatellite marker Lca949, all the remaining five markers were located on QTL intervals at genome wide significance level. For each population, 142 to 248 individuals were used. Substantial phenotypic variation among populations ranging from 40.2 g (F2S5 and F2S6) to 65.2 g (F2S1) was observed at the age of 3 months (BW3M). This variation can result from uncontrolled environmental factors; each population was therefore treated separately during association analysis. Summary description of the association study in the four populations using selected markers is presented in Table 2.

**Table 2:** Significant associations between body weight and candidate markers in the four populations of Asian seabass

**Population ID**	**Individual number**	**Marker name, linkage group and position (cM) in linkage map**					
		**IFABP-a SNP1245**	**IFABP-a SNP1550**	**Lca 949**	**Lca 1022**	**Lca 330**	**Lca Te0095**
		**LG5-0**	**LG5-0.2**	**LG7-8.9**	**LG9-5.9**	**LG21-0**	**LG24-0**
F2S1	142					C*	
						D*	
F2S5	245	A***	A**		A**	A***	A*
		B*	C***		C***	B***	
			D*			C***	
						D***	
F2S6	246	C*					
		D*					
F2S7	248						C*
							D*

Analysis model and data type: A, GLM model with genotype data; B, MLM model with genotype data; C, GLM model with haplotype data; D, MLM model with haplotype data. Significant level: *, *P* < 0.05; **, *P* < 0.01; and ***, *P* < 0.001.

**Figure 3:**
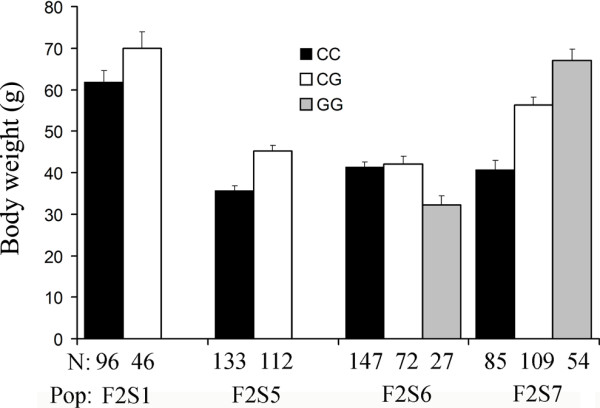
**Differences of observed body weight traits among IFABP-a genotypes in 4 populations.** The mean and standard deviation for body weight at the age of 3 months and the individual number (N) for each of the three genotypes (CC, CG, GG) in each population (Pop) were provided.

The LD association was tested between the homozygous/heterozygous genotypes and haplotypes of the selected markers and phenotype data (BW3M). For each selected marker, an association analysis was performed. An important concordance was observed between QTL mapping and association studies. Except the marker Lca949, the remaining five markers show significant association with body weight trait in one to two populations. Five markers show significant association in population F2S5. The remaining three populations were associated to one or two markers under different models. This may result from the limited population sizes, age differences, and some genotypic combinations of the two loci are unfavorable under some specific conditions. In the four populations, the observed phenotypic data for the heterozygosity genotype (CG) for marker IFABP-a-SNP1245 were generally larger than those for CC genotypes (Figure 3), indicating the SNP has a significant additive effect on BW3M. IFABP-a-SNP1245 haplotypes and genotypes were significantly associated to BW3M in two populations (F2S5 and F2S6). However, to our surprise, when compared the trait data for three genotypes (CC, CG and GG) in F2S6 and F2S7, the GG genotype showed negative effect in F2S6, but showed positive effect in F2S7. These results indicated there were unfavorable factors that associated to G haplotype in F2S6 populations, which also compromised the CG heterozygosity effect in the population. Our study showed that the markers located on the QTL intervals at the genome wide significance level were quite reliable for application in MAS. Since family/population-specific QTL exist, further studies using more markers near QTL and a larger number of families with high performances are required to identify marker-trait associations.

### Characterization of IFABP genes in the Asian seabass

**Figure 4:**
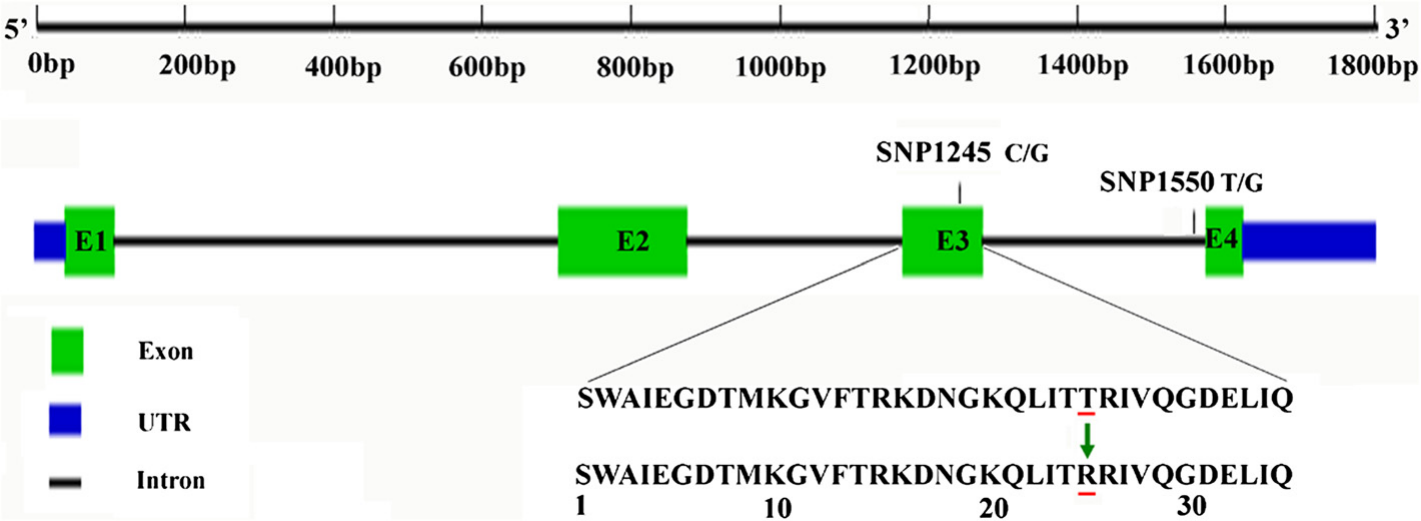
**Exon–intron structure of the IFABP-a gene and position of known SNP polymorphisms.** The 1803 bp genomic sequence, intron/exon structure, two SNP positions and amino acid changes in exon 3 of the IFABP-a gene were presented.

Both QTL and association mapping revealed the IFABP-a SNP1245 marker was significantly associated to growth traits, which explained 5.4-10.5% of phenotypic variance. Therefore, IFABP-a may be an important candidate gene for growth. To further validate its functions in growth, we studied the IFABP gene by cloning and characterization. In fish, IFABP gene was characterized only in few species, e.g., *Paralichthys olivaceus*[34] and zebrafish [35-39]. The IFABP gene shows binding specificity for long-chain fatty acids and is proposed to be involved in uptake of dietary fatty acids and their intracellular transport [35], similar to the roles in mammals. We found that IFABP gene has two isoforms, IFABP-a and IFABP-b, in the Asian seabass. These two isoforms locate on LG5 and LG14, respectively. We cloned the complete ORFs and partial UTRs for both genes. The two isoforms showed similar gene structures, including 4 exons and 3 introns with a deduced polypeptide sequence of 132 amino acids in each gene, similar to that of *P. olivaceus*[34]. However, the two genes are far apart with a genetic distance of 0.278 between the amino acid sequences of two isoforms. The phylogenetic tree analysis suggests that the two seabass IFABP protein genes belong to two distinct groups (Additional file 5: Figure S2). The patterns of diversification suggest a long evolutionary history for the two isoforms. The SNP mutation IFABP-a-SNP1245 showing significant association to growth was located at exon 3 of the gene (Figure 4). In the QTL mapping and association populations, two alleles (C and G) were detected at SNP1245. This C to G transversion caused the amino acid Threonine (Thr/T) changing to Arginine (Arg/R) in the polypeptide sequence. This mutation might have influence on growth in fish. Another SNP IFABP-a-SNP1550 was mutated at the 3rd intron between exon 3 and exon 4 of the gene without significant association to growth (Figure 4).

### Expressions of the two IFABP homologs in the fish intestines in response to fast challenge

**Figure 5:**
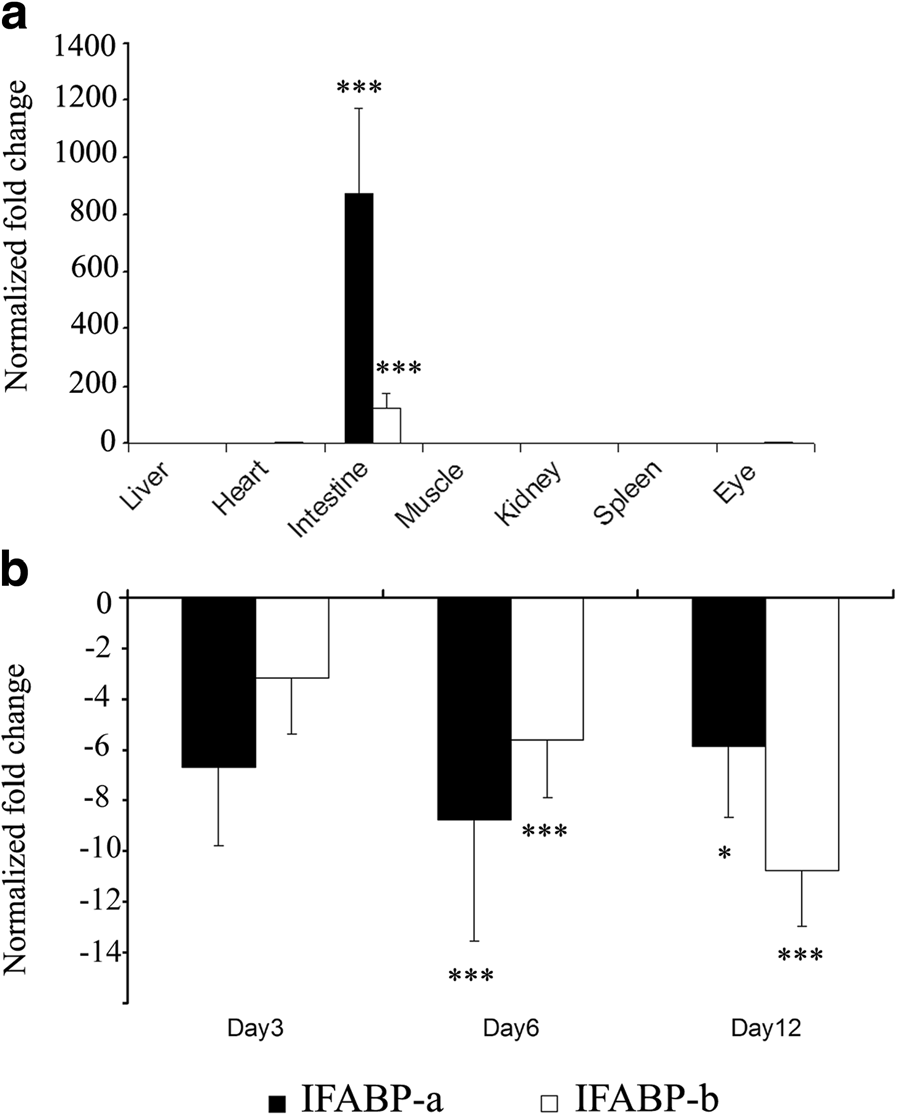
**Temporal and spatial expression of IFABP genes in the Asian seabass revealed by qPCR.** ‘**a**’, both isoforms were abundantly expressed in the intestines, but weakly or barely detectable in the remaining seven tissues; ‘**b**’, temporal expression of the two isoforms in the intestines showed substantial down-regulation at 3, 6, 12 days post fast. ‘*’, a significant level; *p* < 0.05; ‘***’, a significant level, *p* < 0.001.

qPCR revealed that both isoforms were abundantly expressed in the intestines but weakly or barely detectable in other tissues (*P* < 0.001; Figure 5-a). IFABP-a was more highly expressed than IFABP-b in the control intestines (~7 fold). Temporal expression of the two genes in the intestines showed substantial down-regulation at 3, 6, 12 days post fast (5.9-8.8 fold for IFABP-a, and 3.2-10.8 fold for IFABP-b) (Figure 5-b), underlining the functional importance in nutritional digestion and absorption-related pathways of the fish. The IFABP-a gene shows significant down-regulation in samples at 6 days post fast (*P* < 0.001; Figure 5-b), and the IFABP-b shows significant down-regulation in samples at 12 days post fast (*P* < 0.001; Figure 5-b), suggesting different functions for these isoforms. In mammals, significant associations among fatness, the abundance of IFABP and growth [40,41] and common variants showing an important effect on fatness and growth [42-44] were reported. These studies suggested that the growth and fatness shared partially genetic architecture. In our study, QTL mapping and association analysis suggest that IFABP-a gene might regulate the growth in fish. Although direct functional evidence relating the variant to growth was lacking, analyses of IFABP in the Asian seabass by qPCR gave additional support to our QTL findings. Since IFABP gene plays key roles in metabolism though the digestion and absorption of fatty acids in animals, it is reasonable to consider that IFABP-a may be a strong candidate for the important growth genes in fish.

## Conclusion

A genome-wide QTL study was performed for growth traits at age of 6 and 9 months post hatch in the Asian seabass. Twenty-one significant QTL and 24 suggestive QTL were detected across all traits, which might be representative of the genetic architecture of growth traits in the Asian seabass. QTL mapping and association analysis revealed that IFABP-a-SNP1245 was significantly associated to growth. Characterization of IFABP gene gives additional support to our QTL findings and suggests that IFABP-a is strong candidate gene for growth in fish. Our data supply a basis for fine mapping QTL, marker-assisted selection and further detailed analysis of the functions of the IFABP gene in fish growth.

## Methods

### Constructing mapping populations and phenotyping

To construct an F_2_ population for mapping QTL affecting growth traits, an F_1_ population was first constructed by mass crossing of 50 wild Asian seabass that captured from Southeast Asia (Thailand, Indonesia, Malaysia and Singapore). Two F_1_ individuals selected as F_2_ parents based on their performance difference in growth traits were then intercrossed to generate the F_2_ mapping family (F2S2), which contained 359 full sib individuals. The F_2_ individuals were raised communally in a 20-ton indoor tank and maintained on standard feeding regimes until 270 days post hatch. All individuals were tagged at the age of 3 months with microchips (Opulent, Singapore). Phenotypic data including body weight, standard length and total length were recorded on each fish of the family at the age of 6 and 9 months (Table 1), using methods described in Fishbase (http://www.fishbase.org/Identification/Morphometrics/centimeters/Index.php). Fin samples were collected from each fish and kept at −80°C for DNA isolation.

For LD-based association mapping, four F_2_ populations (F2S1, F2S5, F2S6, F2S7) were used. Each population was established from mating of unrelated 25 F_1_ sires with 25 F_1_ dams, whose parents originated from natural populations in Southeast Asian. The genetic relatedness and genetic distances among individuals were evaluated by using software PAPA [45] and MEGA 4 [46]. Only unrelated individuals were used in mass crosses. Parentage assignment for each population was conducted to reconstruct pedigrees. Each population containing around 2000 fishes was transferred to a 7-ton indoor tank. The fishes were fed with commercial dry feed. After three months of growing in the tanks, two extreme trait groups (large size group: 60–200 fishes, and small size group: 60–100 fishes) from each tank were sampled and phenotypic data from each fish were measured. All experiments in this work have been reviewed and approved by the IACUC Committee on Bioethics of the Temasek Life Sciences Laboratory, Singapore.

### Microsatellite and SNP genotyping

Based on the Asian seabass linkage maps [15,16], 123 microsatellite markers shown polymorphism in parents of the mapping family (F2S2) were selected for genotyping analysis. These microsatellite markers distributed evenly on 24 linkage groups and were genotyped on a 3730xl DNA analyzer (Applied Biosystems) after PCR amplification using fluorescently labeled primers. The two parents and their offspring (N = 359) were genotyped using microsatellites as described previously [15].

Based on our Asian seabass transcriptome database (unpublished data), 19 ESTs showing high similarity to genes related to growth, nutritional condition or environmental adaptation were aligned with genomic sequence data from Stickleback (*Gasterosteus aculeatus*; http://www.ensembl.org/Gasterosteus_aculeatus/Info/Index). Primer sites in conserved exon regions were identified. One or two primer pairs for each gene (see Additional file 1: Table S1) were developed based on the EST sequences using the program PrimerSelect (DNASTAR, Wilmington, DE). Genotypes of SNPs in candidate genes were detected by sequencing of PCR products. SNP genotype was analyzed by using software Sequencher 4.9 (Gene Codes, Ann arbor, USA). To design primers for real-time PCR, the exon/intron boundaries and divergence regions from two IFABP homologs were first identified by using the software Sequencher 4.9. One primer pair that spans exon/intron boundary on the mRNA for each homolog was designed using the program PrimerSelect (DNASTAR, Wilmington, DE). This primer design allows differentiating the two homlogs and doesn’t amplify genomic DNA.

### Fasting experiment and sampling

Twenty-eight fishes (at ~ 1 year old) that maintained at the fish facility of Temasek life sciences laboratory were used for functional analysis. Prior to fast experiment the fishes were housed in one tank containing 2000 L of freshwater. On day 0 (starvation day 0), seven tissues (kidney, heart, muscle, intestine, spleen, eye and liver) from four fishes were taken and kept in Trizol reagent. The remaining twenty-four fishes were then transferred to two tanks holding 1000 L of freshwater. The twelve fishes in control tank were fed with commercial dry feed twice per day. In test tank, a fast regime was imposed on twelve fishes for up to 12 additional days under the same conditions as the control except without feeding. Four fishes from each tank were sacrificed on days 3, 6 and 12 of the fast period. Intestine samples were kept in Trizol reagent (Invitrogen, CA, USA) for RNA extraction.

### Cloning and gene expression analysis of the IFABP gene by quantitative real-time PCR (qPCR)

The full-length cDNA libraries described as in Xia et al. [47,48] was used for cloning and characterization of the IFABP gene. For analysis of gene expression patterns 10 times dilution of the cDNA were assayed as template by qPCR using EF1A and RPL13A as control (see Additional file 1: Table S1), as suggested by Tang et al. [49] that the EF1A and Rpl13A genes are more suitable as a reference gene panel for zebrafish tissue analysis. PCR reactions were performed with the iQ SYBR Green Supermix (Bio-Rad, Hercules, CA, USA) as described by the manufacturer in an iQ™5 Real Time PCR Detection Systems (Bio-Rad, Hercules, CA, USA). PCRs were performed in four biological replicates and three technical replicates. For the analysis of the changes of gene expression, the values of qPCR reactions were normalized to EF1A and RPL13A gene expression, calculated by the ΔΔCT method [50] as implemented by the software Bio-Rad iQ5 (2.0) (Bio-Rad, Hercules, CA, USA). *P*-value significant difference was calculated using the student’s *T*-Test module installed in the Microsoft Office Excel 2008 program with parameters setting as two-tailed distribution and two-sample equal variance.

### Data analysis

#### Linkage and QTL analysis

A sex averaged genetic linkage map was generated for the full sib family containing 359 individuals using JoinMap linkage analysis software packages [51]. A LOD score of 3.0 was used as a threshold value for defining linkage groups. Marker distances were evaluated according to the Kosambi formula (in cM). QTL analyses were performed with MapQTL version 5.0 [52] by using the multiple QTL model mapping analysis with selected cofactor markers. The LOD profiles were generated at 1 cM intervals along each linkage group to identify the most likely QTL position. The linkage group wide and genome wide significance thresholds for LOD scores were determined by implementing a bootstrapping method with 10000 permutation iterations at *p* = 0.01 and 0.05. QTL that exceeded the linkage group-wide LOD threshold at *p* < 0.05 was reported as suggestive QTL, while exceeding a genome-wide LOD threshold of *p* < 0.05 was considered evidence for a significant QTL effect, and exceeding a genome-wide LOD threshold of *p* < 0.01 was considered evidence for a very significant QTL effect. QTL affecting different traits were considered to be located in the same region if the chromosomal positions of these QTL were overlapped. Linkage map and QTL regions affecting growth were visualized using MapChart software (ver. 2.1) [53] and shown in Additional file 3: Figure S1.

#### LD-based association analysis and heterozygosity effect

In association analysis, each of the 4 populations from mass crosses was analyzed separately, as there are differences in the maintenance of each population in separated tanks, which could distort the estimation of association effects if they were analyzed together. The genetic structure of each population was subdivided by using a model-based approach in the STRUCTURE software [54]. Kinship matrices were calculated using the SPAGeDi software package [55]. Association analysis between the haplotypes and genotypes of the markers/genes and growth traits was then carried out using general linear model (GLM) function and mixed linear model (MLM) function that implemented in the software TASSEL 2.0.1 [56].

In order to investigate associations between phenotypic traits and molecular genotypes, two extreme trait groups (N = 30/group) for each trait were selected from 359 full sib fishes from the family F2S2. Excluding the loci with heterozygous genotypes (ab×cd) that segregated in two parents (with 4 alleles), the observed heterozygosities and homozygosities of remaining loci that used in the construction of the linkage groups were calculated for each group. The one-tailed probability for frequency distribution differences of heterozygosities between two extreme groups of a trait was calculated using on line Fisher's exact test calculator (Ver.3; http://www.danielsoper.com/statcalc3/calc.aspx?id=29).

#### Sequence analysis and phylogenetic analysis

The Asian seabass IFABP gene sequences (GenBank accession no.: JX678843-JX678844) were used to perform BLAST analyses for EST annotation. Twenty one IFABP protein sequences, sixteen of which from fish databases, were retrieved from the NCBI database (http://mirrors.vbi.vt.edu/mirrors/ftp.ncbi.nih.gov/blast/db/). The protein sequences were aligned using the Clustal X 1.83 program [57]. Phylogenetic analyses was carried out using the MEGA 4 package [46] with neighbour-joining bootstrap tests (1000 times). Genetic distances among protein sequences were evaluated with Poisson correction by MEGA 4. Evolutionary relationships of IFABP protein genes were shown in Additional file 5: Figure S2.

#### Descriptive statistics and correlation analysis

Basic descriptive statistics, including number of observations (N), minimum values, maximum values, means and standard deviations (sd) and Pearson's correlation coefficient were calculated using the ‘data analysis’ module of the software Excel.

## Abbreviations

QTL: Quantitative trait loci; SNP: Single nucleotide polymorphism; cM: Centimorgan; FABP: Fatty-acid-binding protein gene; IFABP-a: Intestine fatty-acid-binding protein alpha gene.

## Competing interests

The authors declare that they have no competing interests.

## Authors’ contributions

GHY initiated and overviewed the Asian seabass project and finalized the manuscript. JHX and GHY designed the study. JHX, GL, XPH, PL, FS, FL and RJT conducted the experiments. JHX conducted the data analysis and wrote the manuscript. All authors have read and approved the final manuscript.

## Supplementary Material

Additional file 1: Table S1 Primer information for the SNP markers used in linkage mapping and gene expression analysis by quantitative RT-PCR. Click here for file

Additional file 2: Table S2 Summary of the Asian seabass linkage map based on microsatellite and SNP markers. Click here for file

Additional file 3: Figure S1 The averaged linkage map and the positions of QTL affecting growth traits in the F2S2 family of the Asian seabass. Click here for file

Additional file 4: Table S3 Significant heterogeneity of genotypes between two extreme size groups in the full sib mapping family. Click here for file

Additional file 5: Figure S2 Evolutionary relationships of IFABP protein genes. The evolutionary history is inferred using the neighbor-joining method. Bootstrap confidence values are listed to the left of the nodes. The GenBank accession no. and species name for each IFABP protein sequence is given to the right. The two IFABP isoforms that cloned from the Asian seabass are underlined. A scale near the bottom relates the length of a branch to the number of the amino acid substitutions that have taken place along the branch (evolutionary distances). Click here for file
