# Supplementary figures and images for: Whole genome scanning and association mapping identified a significant association between growth and a SNP in the IFABP-a gene of the Asian seabass
Source: BMC Genomics. 2013 May 1;14:295. doi: 10.1186/1471-2164-14-295 (PMC3653795; doi:10.1186/1471-2164-14-295)

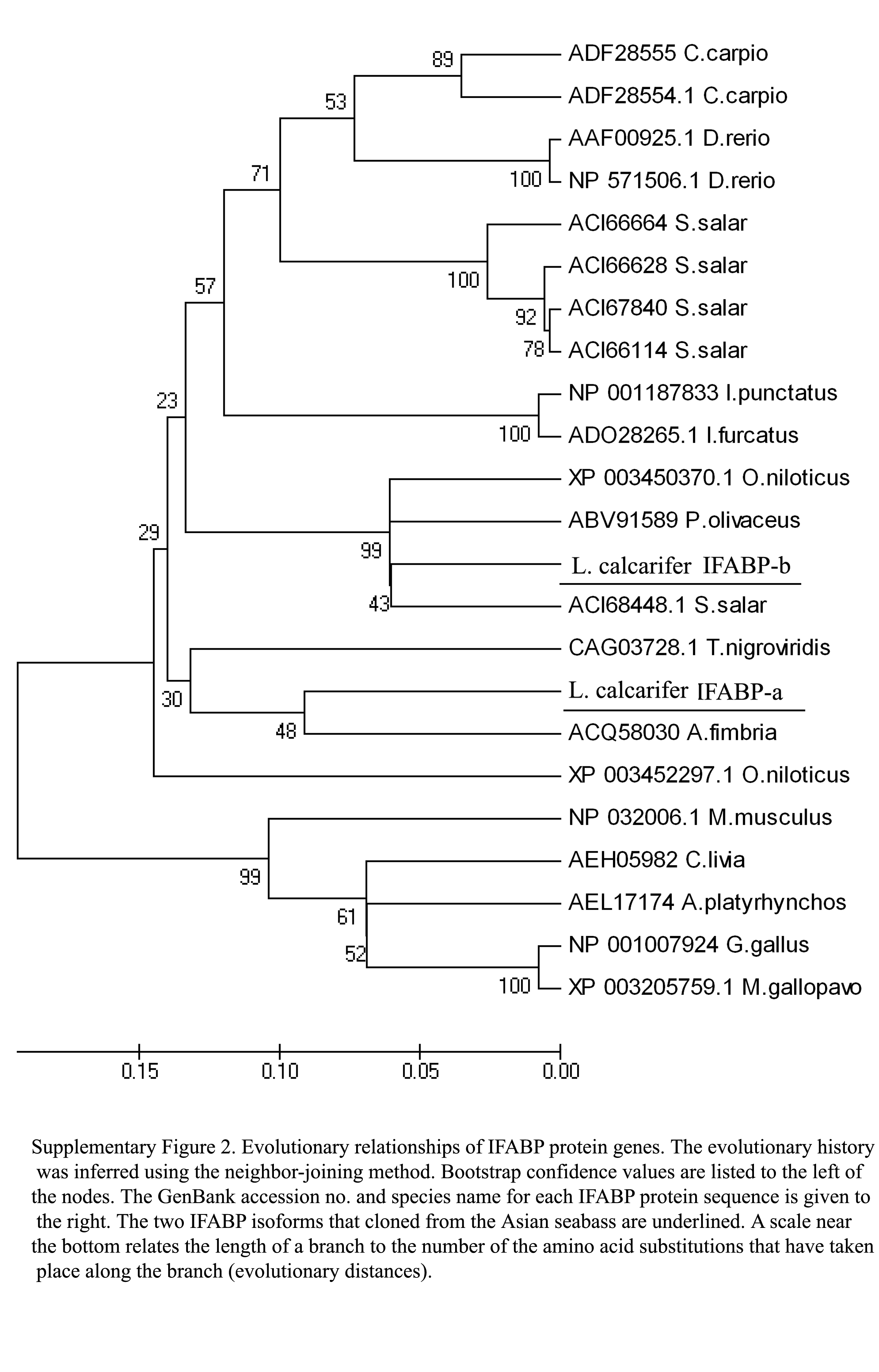

Supplement: Additional file 5: Figure S2 — Evolutionary relationships of IFABP protein genes. The evolutionary history is inferred using the neighbor-joining method. Bootstrap confidence values are listed to the left of the nodes. The GenBank accession no. and species name for each IFABP protein sequence is given to the right. The two IFABP isoforms that cloned from the Asian seabass are underlined. A scale near the bottom relates the length of a branch to the number of the amino acid substitutions that have taken place along the branch (evolutionary distances). [file 1471-2164-14-295-S5.tiff]
