# Supplementary material for: Whole genome scanning and association mapping identified a significant association between growth and a SNP in the IFABP-a gene of the Asian seabass
Source: BMC Genomics. 2013 May 1;14:295. doi: 10.1186/1471-2164-14-295 (PMC3653795; doi:10.1186/1471-2164-14-295)
Supplement: Supplementary file 5 [file 1471-2164-14-295-S3.doc]

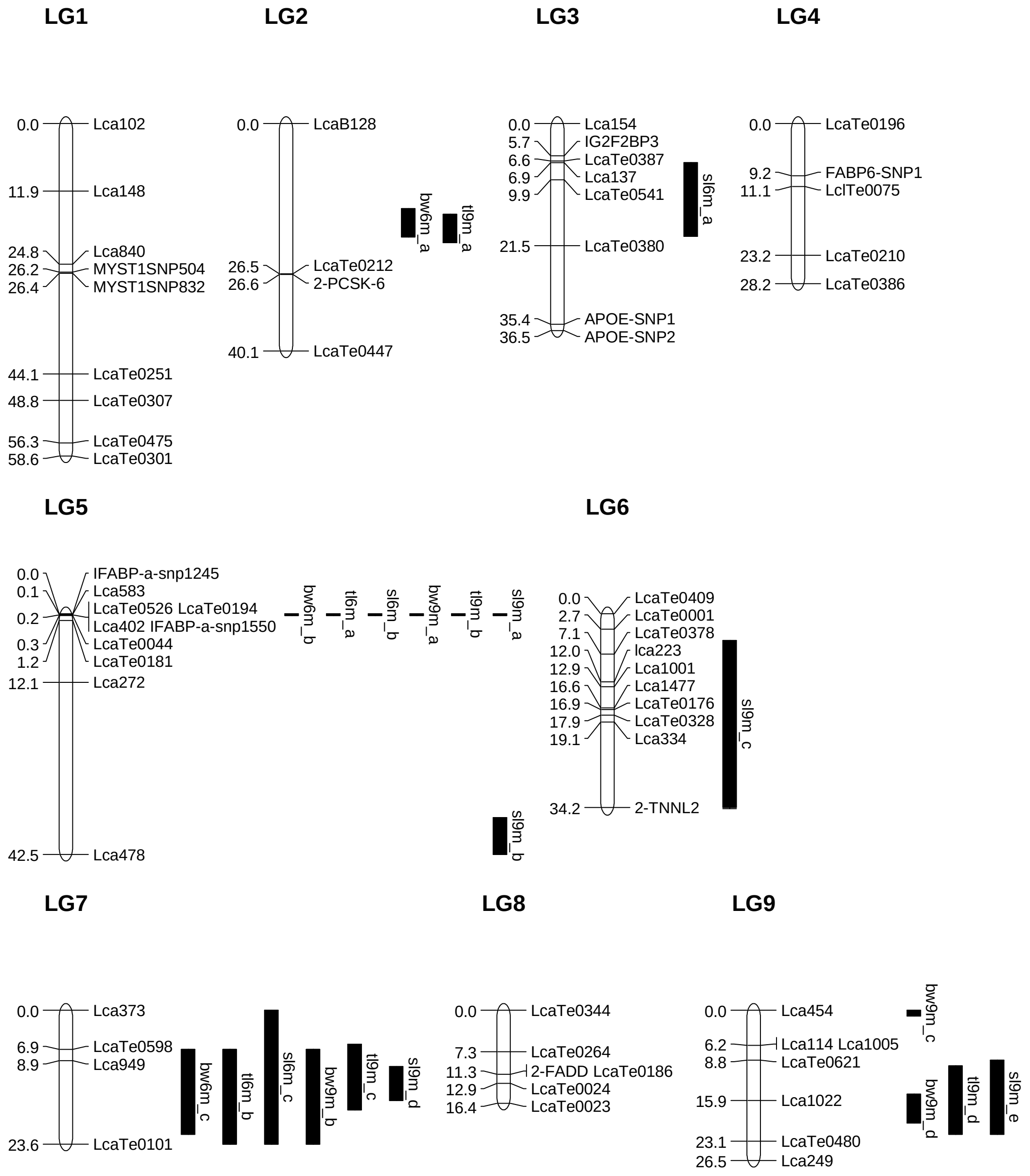

LG1
0.0 Lca102
11.9 Lca148
24.8 Lca840
26.2 MYST1SNP504
26.4 MYST1SNP832
44.1 LcaTe0251
48.8 LcaTe0307
56.3 LcaTe0475
58.6 LcaTe0301
LG2
0.0 LcaB128
26.5 LcaTe0212
26.6 2-PCSK-6
40.1 LcaTe0447
bw6m_a
tl9m_a
LG3
0.0 Lca154
5.7 IG2F2BP3
6.6 LcaTe0387
6.9 Lca137
9.9 LcaTe0541
21.5 LcaTe0380
35.4 APOE-SNP1
36.5 APOE-SNP2
sl6m_a
LG4
0.0 LcaTe0196
9.2 FABP6-SNP1
11.1 LclTe0075
23.2 LcaTe0210
28.2 LcaTe0386
LG5
0.0 IFABP-a-snp1245
0.1 Lca583
0.2 LcaTe0526 LcaTe0194
Lca402 IFABP-a-snp1550
0.3 LcaTe0044
1.2 LcaTe0181
12.1 Lca272
42.5 Lca478
bw6m_b
tl6m_a
sl6m_b
bw9m_a
tl9m_b
sl9m_a
sl9m_b
LG6
0.0 LcaTe0409
2.7 LcaTe0001
7.1 LcaTe0378
12.0 lca223
12.9 Lca1001
16.6 Lca1477
16.9 LcaTe0176
17.9 LcaTe0328
19.1 Lca334
34.2 2-TNNL2
sl9m_c
LG7
0.0 Lca373
6.9 LcaTe0598
8.9 Lca949
23.6 LcaTe0101
bw6m_c
tl6m_b
sl6m_c
bw9m_b
tl9m_c
sl9m_d
LG8
0.0 LcaTe0344
7.3 LcaTe0264
11.3 2-FADD LcaTe0186
12.9 LcaTe0024
16.4 LcaTe0023
LG9
0.0 Lca454
6.2 Lca114 Lca1005
8.8 LcaTe0621
15.9 Lca1022
23.1 LcaTe0480
26.5 Lca249
bw9m_c
bw9m_d
tl9m_d
sl9m_e

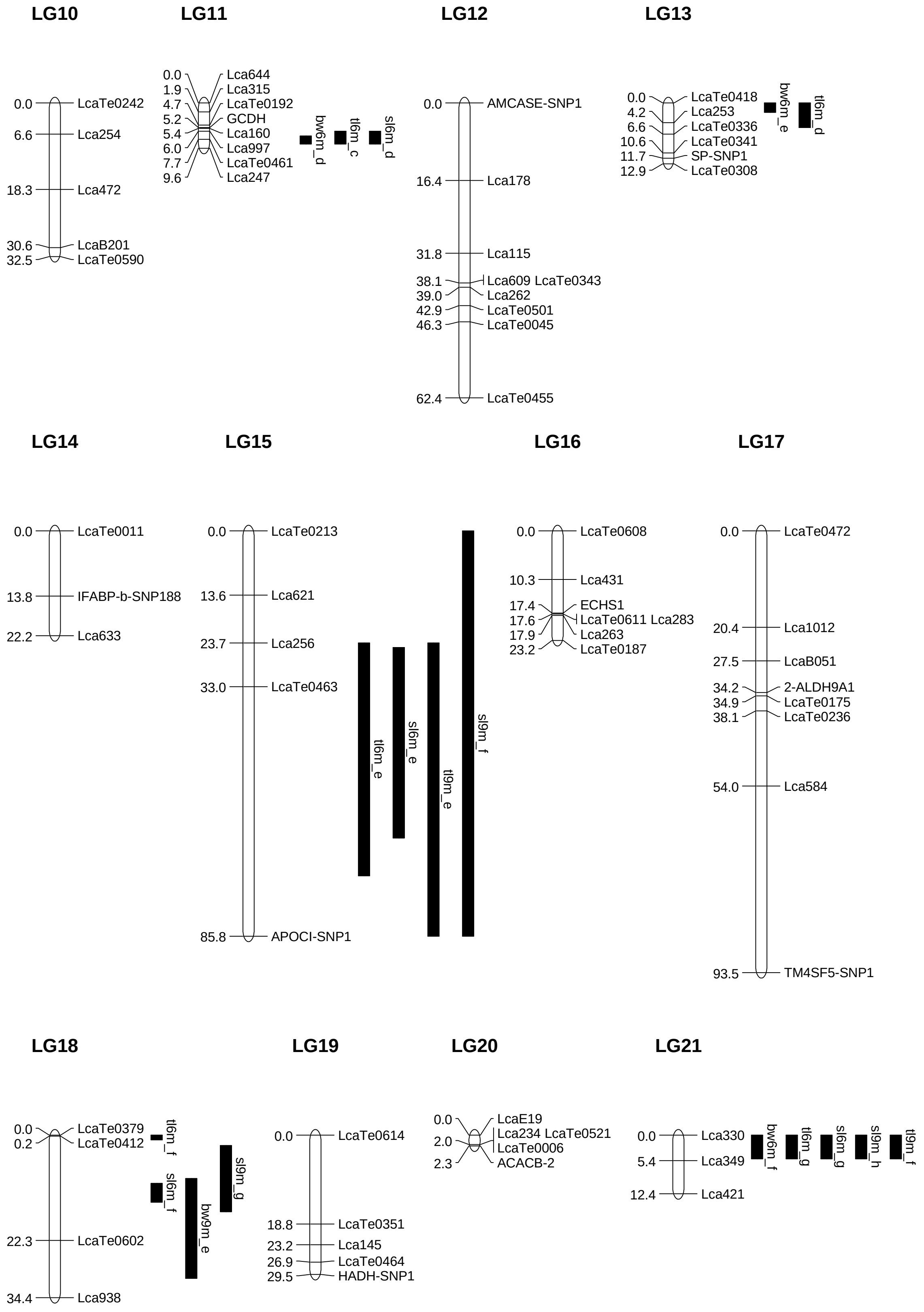
LG10
0.0 LcaTe0242
6.6 Lca254
18.3 Lca472
30.6 LcaB201
32.5 LcaTe0590
LG11
0.0 Lca644
1.9 Lca315
4.7 LcaTe0192
5.2 GCDH
5.4 Lca160
6.0 Lca997
7.7 LcaTe0461
9.6 Lca247
bw6m_d
tl6m_c
sl6m_d
LG12
0.0 AMCASE-SNP1
16.4 Lca178
31.8 Lca115
38.1 Lca609 LcaTe0343
39.0 Lca262
42.9 LcaTe0501
46.3 LcaTe0045
62.4 LcaTe0455
LG13
0.0 LcaTe0418
4.2 Lca253
6.6 LcaTe0336
10.6 LcaTe0341
11.7 SP-SNP1
12.9 LcaTe0308
bw6m_e
tl6m_d
LG14
0.0 LcaTe0011
13.8 IFABP-b-SNP188
22.2 Lca633
LG15
0.0 LcaTe0213
13.6 Lca621
23.7 Lca256
33.0 LcaTe0463
85.8 APOCI-SNP1
tl6m_e
sl6m_e
tl9m_e
sl9m_f
LG16
0.0 LcaTe0608
10.3 Lca431
17.4 ECHS1
17.6 LcaTe0611 Lca283
17.9 Lca263
23.2 LcaTe0187
LG17
0.0 LcaTe0472
20.4 Lca1012
27.5 LcaB051
34.2 2-ALDH9A1
34.9 LcaTe0175
38.1 LcaTe0236
54.0 Lca584
93.5 TM4SF5-SNP1
LG18
0.0 LcaTe0379
0.2 LcaTe0412
22.3 LcaTe0602
34.4 Lca938
tl6m_f
sl6m_f
bw9m_e
sl9m_g
LG19
0.0 LcaTe0614
18.8 LcaTe0351
23.2 Lca145
26.9 LcaTe0464
29.5 HADH-SNP1
LG20
0.0 LcaE19
2.0 Lca234 LcaTe0521
LcaTe0006
2.3 ACACB-2
LG21
0.0 Lca330
5.4 Lca349
12.4 Lca421
bw6m_f
tl6m_g
sl6m_g
sl9m_h
tl9m_f

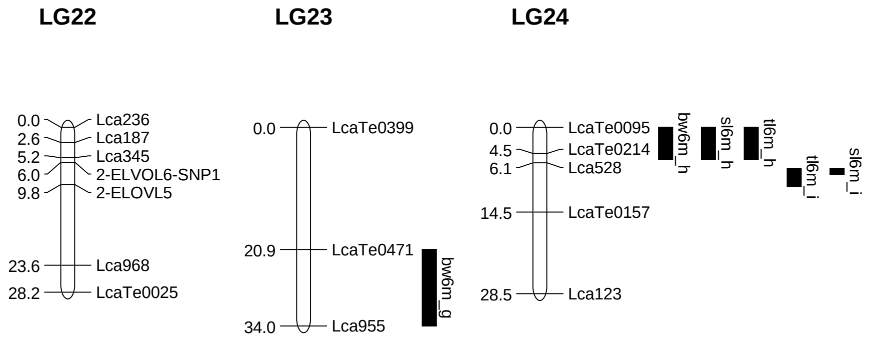
LG22
0.0
2.6
5.2
6.0
9.8
23.6
28.2
Lca236
Lca187
Lca345
2-ELVOL6-SNP1
2-ELOVL5
Lca968
LcaTe0025
LG23
0.0
20.9
34.0
LcaTe0399
LcaTe0471
Lca955
bw6m_g
LG24
0.0
4.5
6.1
14.5
28.5
LcaTe0095
LcaTe0214
Lca528
LcaTe0157
Lca123
bw6m_h
sl6m_h
tl6m_h
tl6m_i
sl6m_i
